# Socio-cultural integration and holistic health among Indigenous young adults

**DOI:** 10.1186/s12889-022-13395-3

**Published:** 2022-05-18

**Authors:** Melissa Walls, Dane Hautala, Ashley Cole, Lucas Kosobuski, Nicole Weiss, Kyle Hill, Stephanie Ozhaawashkodewe’iganiikwe Williams

**Affiliations:** 1 Johns Hopkins University Bloomberg School of Public Health, Center for American Indian Health, Baltimore, USA; 2 Oklahoma State University, Stillwater, USA; 3 University of Minnesota, Minneapolis, USA; 4 University of North Dakota, Grand Forks, USA; 5 White Earth Nation, White Earth, USA

**Keywords:** Socio-cultural integration, Social support, Social integration, Indigenous, American Indian, First Nations, Wellbeing

## Abstract

**Background:**

Research on associations between social integration and wellbeing holds promise to inform policy and practice targets for health promotion. Yet, studies of social connection too frequently rely on overly simplistic measures and give inadequate attention to manifestation and meanings of social integration across diverse groups. We use the term *socio-cultural integration* to describe expanded assessment of both social and cultural aspects of belonging and connection.

**Methods:**

We examined 7 distinct indicators of socio-cultural integration, identified heterogeneous patterns of responses across these indicators using latent profile analysis, and determined their relevance for wellbeing using survey data from a study with Indigenous communities in the U.S. and Canada. Wellbeing was measured using holistic ratings of self-rated physical, emotional, and spiritual health.

**Results:**

Latent profile analysis (LPA) of responses to the 7 socio-cultural integration variables yielded a 3-class model, which we labeled low, moderate, and high integration. Mean scores on self-rated physical, mental and spiritual health were significantly associated with LPA profiles, such that those in the low integration group had the lowest self-rated health scores and those in the high integration group had the highest health scores. With the exception of similar ratings of cultural identification between low and moderate integration profiles, patterns of responses to the diverse socio-cultural integration measures varied significantly across the 3 latent profiles.

**Conclusions:**

Results underscore the importance of expanding our assessment of social integration with attention to the interrelationships of family, community, culture, and our environment. Such concepts align with Indigenous conceptions of wellbeing, and have relevance for health across cultures. More concretely, the indicators of socio-cultural integration used in this study (e.g., cultural identity, having a sense of connectedness to nature or family, giving or receiving social support) represent malleable targets for inclusion in health promotion initiatives.

## Background

Connection and belonging are crucial components of human life such that broad indicators of social integration including positive social influences, sense of mattering, companionship, and perceptions of support have been linked to improved physical and mental health [1, 2]. In a review of prior research, Holt-Lunstad and colleagues [3] identified three commonly measured dimensions of social connectedness: structural (connections across domains/roles), functional (provisions or perceptions of support), and quality (appraisal of positive/negative influences of support systems), noting that each of these domains has been linked to reductions in mortality. Continued research on social integration and wellbeing thus holds promise to inform policy and practice targets for health promotion. Such a focus provides a balanced, strengths-based counter-narrative to the proliferation of attention to isolation and loneliness, constructs of profound health consequence with effects comparable to risks associated with smoking, physical inactivity, and hypertension [4, 5].

Yet, existing studies of social connection too frequently rely on simplistic measures despite its inherently complex, multi-dimensional nature [1, 4, 6, 7]. A 2010 meta-analysis of the effects of social connections on mortality revealed that more sophisticated measures of social relationships, such as social integration, were stronger predictors of survival than were simplistic indicators (e.g., binary indicators of living alone; single-item measures like relationship status; assessment of single types of relationships) [4]. A related shortcoming of extant research is inadequate attention to manifestation and meanings of social integration across diverse groups. This is critical because studies in cross-cultural settings can reveal distinctions and commonalities useful for population health promotion. Furthermore, cultural contexts both shape and are shaped by experiences with loneliness and social integration [8, 9]. Analyses by Beller and Wagner (2020) [10] found that cultural individualism moderated relationships between loneliness and poorer health, with stronger associations observed in more collectivist countries/societies. Distinct dimensions of social integration may also vary in meaning and impact by culture (i.e., the knowledge, behaviors, beliefs, and values of groups across society). For instance, providing social support to others can bolster a sense of purpose within one’s community [1, 11], a process that may be more influential in communally-oriented groups. Overall, it is likely that current evidence on the benefits of socio-cultural integration for health (which heavily relies on Euro-centric, more individualistic cultures and samples) may, in fact, be conservative appraisals of true effects [10]. Studying social integration across cultures, and with cultural frameworks of wellbeing in mind, may confer more accurate conclusions to promote wellbeing for all [12]. As such, we use the term socio-cultural integration to describe expanded assessment of both social and cultural aspects of belonging and connection.

Research with and by Indigenous communities is uniquely positioned to address limitations of prior research and provide lessons regarding socio-cultural integration and its utility in combatting health inequities. Several factors underscore this point. First, connectedness is a critical basis of Indigenous worldviews, including among original inhabitants of the North American continent (i.e., the hundreds of American Indian, First Nations, Inuit, Métis, and Alaska Native groups in the U.S. and Canada). Theories and models of Indigenous wellbeing emphasize relationships and interdependence among individuals, families, communities, the natural world, spirit, and culture [13]. As examples, the Relational Worldview theory describes interrelationships and balance across four domains of individual wellness, including: context, mind, body, and spirit [14, 15]. The Cultural Continuity Model [16] posits that individuals need to persist as authentic selves (self-continuity) for healthy physical, emotional, and cognitive development, and this concept of self-continuity extends to cultural continuity. The latter relates to social changes within Indigenous communities and abilities to connect traditional pasts with contemporary and future contexts [16].

Second, traditional Indigenous cultural ways and sense of community have been disrupted by colonization, and health inequities are extreme, underscoring the urgency of community driven, culturally relevant public health research and action with Indigenous communities. Historical trauma as a result of the colonization of Indigenous Peoples represents historically anchored, intergenerational, and ongoing wounds resulting from mass group trauma perpetuated with purposeful intent [17, 18]. Historical trauma and cultural loss have been associated with feelings of isolation and the breakdown of relationships, community ties, kinship networks, and relationships to traditional lands by way of dispossession [19, 20]. For many Indigenous communities, health involves “needing to be part of the larger family and community body,” wherein disconnection is linked to illness from the outside world [21]. These and other historically rooted social determinants of health are widely viewed as fundamental causes of Indigenous health inequities [22], most strikingly illustrated by the fact that American Indian and Alaska Native peoples experience the highest rates of premature mortality in the US compared to any other racial/ethnic group [23].

Third, despite this harrowing history and inequitable health outcomes, Indigenous values of interconnectedness persist and are a critical feature of survival in the face of colonization [13] alongside reclamation of cultural values and practices [24–26]. In short: protective factors are enduring and prominent within Indigenous communities. Relatedly, tribal communities have long called for culturally grounded approaches to redressing health inequities and reviving socio-cultural connections [25]. A result is community-driven innovations in the assessment of protective factors, conceptual models, and strengths-based approaches to wellbeing. For instance, a novel measure of Alaska Native Peoples’ awareness of connectedness has been empirically linked to protection from suicidality and alcohol abuse [27–29]. Research with Indigenous Peoples’ demonstrates how cultural involvement enhances the effectiveness of medical treatments and facilitates healing; this effect may be attributed to the sense of identity found in collectivistic cultures, traditional cultural practices, and establishing a sense of belonging or purpose in the world [24]. First Nations communities in Canada that have taken active steps to preserve and rehabilitate their cultures have demonstrated lower rates of suicide among youth [16]. Qualitative research in Alaska describes how Indigenous Elders view connection to culture as a linkage to “something intergenerational and therefore larger than themselves,” [30] thus fueling a sense of belonging and purpose critical to wellbeing. In the same study, Alaska Native youth talked about feeling connected to community and culture by listening to traditional stories from Elders [30]. In these ways, Indigenous communities hold clues for promoting and reviving social connections important for strength-based approaches to health equity and health promotion for all cultures.

We posit that the interconnectedness of both social and cultural integration is more salubrious than they appear as individual constructs. High levels of integration across social and cultural domains are expected to be associated with greater perceptions of self-rated health compared to lower levels or inconsistent levels (e.g., high levels on some constructs, low on others) of socio-cultural integration. As such, the aim of this study is to examine 7 distinct indicators of socio-cultural integration, identify heterogeneous patterns of responses across these indicators using latent profile analysis, and determine their relevance for wellbeing using data from a longitudinal study with Indigenous communities in the U.S. and Canada. Given the lack of prior work and exploratory nature of this study, no specific hypotheses were proffered.

## Methods

### Study design

Data are from *Healing Pathways*, a longitudinal community-based participatory research (CBPR) project, which began in 2002, at which time all tribally enrolled adolescents aged 10–12 years living on or near (within 50 miles) the reservation/reserve were invited to participate along with up to two adult caregivers. Participants were interviewed annually for 8 years from 2002 – 2010. In 2016, we began a ninth wave of interviews with the original study target adolescents, who by then were young adults. More detailed descriptions of the CBPR orientation and methodology for Healing Pathways are viewable elsewhere [31–33]. Briefly, our approach centers Indigenous self-determination, leadership and relationship-focused team co-creation in the research process (for example, the PI for the project [MW] is Anishinaabe). As a team, we secure formal Tribal Council/Board permissions prior to applying for project funding. We work collaboratively as university-based and community based team members; the latter includes formalized project boards called Community Research Councils (CRCs) consisting of tribal community members of varying ages, work experiences, and perspectives. Together, we make decisions about survey design and specific survey questions, data collection team hiring and personnel decisions, set project priorities, and complete community-based and scientific project findings dissemination activities (e.g., presentations to Tribal Leaders, co-presentations at academic conferences, collaborative publishing, development of infographics, etc.). All manuscripts written using Healing Pathways data are shared with CRCs for formal feedback and dialogue about results prior to submission to academic journals. Five (MW, AC, LK, KH, SW) of the seven lead authors for the current study are Indigenous scholars and/or community members.

### Setting

Data for the Healing Pathways study come from four reservations in the Northern Midwest US and four Canadian First Nations reserves; all representing a single Indigenous cultural group.

### Sample

The sample for the current analyses includes 513 participants who completed wave 10 interviews. We focus on wave 10 because this was the first wave for which we included expanded assessments of socio-cultural integration in surveys. Wave 10 data was collected from 2018–2019 (we experienced a lag in study funding and data collection between waves 8 and 9; hence the gap in assessment timing) when participants were aged 26–28 years (Mean Age = 27.8 years). Participants received $50 as an incentive for their participation.

### Ethical considerations

All participants were engaged in a process of consent that included signed informed consent procedures. Study methods were/are collaboratively developed by community and university team members and approved by the University institutional review boards, and all methods were carried out in accordance with relevant guidelines and regulations. Results are also shared with communities and participants via technical reports, infographics, project websites, and a project YouTube channel [34, 35].

### Data collection

#### Instruments

We assessed seven measures of social and cultural connection. Collectively, these measures represent indicators of socio-cultural integration. All measures were selected or created, reviewed, and where necessary, piloted and revised in collaboration with CRC members to maximize local and cultural validity, comprehension, and utility. Each of the socio-cultural integration measures have been used by our team in prior research with Ojibwe adults living with type 2 diabetes.

##### Social support

Social support was assessed using an adapted version of the 2-Way Social Support Scale developed by Shakespeare-Finch & Obst [36]. In the original formation and validation of the scale, factor analysis models showed that receiving emotional support, giving emotional support, receiving instrumental support, and giving instrumental support loaded onto separate factors. In this study, using principle axis factor analysis, all receiving social support indicators loaded onto one factor, and all giving social support indicators loaded onto another (two factor eigenvalues > 1; oblique rotation factor loadings > 0.55). For receiving social support, respondents were asked how much they agreed with six statements about receiving social support (e.g., there is someone in my life I can get emotional support from, I have someone to help me if I am physically unwell). For giving social support, respondents were asked eight questions about giving social support (e.g., when someone I was close to was sick I helped them, I give others a sense of comfort in times of need). Response options ranged from (0) not at all true to (3) always true. Items were summed to create a scale of *receiving social support* (α = 0.91) and *giving social support* (α = 0.86).

##### Social connection

Loneliness is a construct that taps into “weak social ties” and was assessed using an adapted version of the UCLA Loneliness Scale [37]. To be consistent with the other scales, all items were reverse coded, so that higher scores corresponded with lower levels of loneliness. Respondents were asked six questions about loneliness (e.g., I have nobody to talk to, I feel completely alone). Response options ranged from (0) always to (3) never. Items were summed to create a measure of *social connection* (α = 0.91).

##### Multicultural mastery

Multicultural mastery was assessed with the Multicultural Mastery Scale developed by Fok and colleagues [38]. Respondents were asked six questions about their friends and family (e.g., working together with friends/family, I can solve my problems). Response options ranged from (0) strongly disagree to (3) strongly agree. Separate multicultural mastery scales were created for peers (α = 0.86) and family (α = 0.89) by summing items together.

##### Cultural identification

Cultural identification was assessed with seven questions from the In-Group Identification scale developed by Leach and colleagues [39]. Respondents were asked how much they agreed or disagreed with seven items about being Native (e.g., I feel a bond with other Native people, I feel committed to Native people). Response options ranged from (0) strongly disagree to (3) strongly agree. The items were summed to create a scale of *cultural identification* (α = 0.89).

##### Awareness of connectedness

Connectedness was assessed with the Awareness of Connectedness Scale developed by Mohatt and colleagues [29]. Connectedness to community, nature, and family were assessed with two items each, in which participants were asked how much they agree with each statement (e.g., I feel connected to nature). Principle axis factor analysis showed that all six items loaded onto one factor (only one factor had an eigenvalue > 1, and oblique rotation factor loadings for each indicator were > 0.45) rather than factoring out into three separate constructs. All six items were summed to create a measure of *awareness of connectedness* (α = 0.75).

##### Outcomes: self-rated health

Wellbeing is measured using holistic ratings of self-rated physical, emotional, and spiritual health based on calls for researchers to consider a range of health outcomes in studies of social isolation/integration [40]. Respondents were asked to rate their overall physical, mental, and spiritual health. Response options were (0) poor, (1) fair, (2) good, (3) very good, and (4) excellent.

##### Demographics

Six demographic variables were examined: Gender (0 = male, 1 = female); age (continuous); personal income (continuous); currently residing on/off reservation (0 = off reservation, 1 = on reservation); education (less than high school [reference group], high school diploma or GED, some college or vocational training, and college degree); and marital/relationship status (single [reference group], married, in a relationship and cohabiting, in a relationship and not cohabiting, and all other relationship statuses).

### Data analysis

Latent profile analysis (LPA) was used to examine heterogeneity across the 7 diverse social and cultural integration measures in Mplus Version 8.1 [41]. LPA aims to identify groups or clusters of individuals who share similar responses across a set of observed indicators (i.e., socio-cultural integration; [42]. These latent groups, or classes, are unobserved and are inferred from the data. Class enumeration is assessed through both statistical fit indices, and the substantive interpretability of the classes. The Akaike information criteria (AIC), Bayesian information criteria (BIC), sample adjusted BIC (SA-BIC), Lo-Mendell-Rubin likelihood ratio test (LMR-LRT; [43], and the bootstrapped likelihood ratio test (BLRT; [44]) were examined as fit statistics to select the optimal number of latent profiles. For the three information criteria (AIC, BIC, and SA-BIC), lower values indicate better model fit, and significant likelihood ratio tests (LMR-LRT and BLRT) indicate that a k-class model fits the data better than a k-1 class model. The three-step approach [45] was used to examine the relationship between demographic variables and latent profile membership. Each demographic variable was entered individually (unadjusted model) and simultaneously with the other variables (adjusted model) in a multinomial logistic regression model predicting profile membership. To examine the relationship between latent profile membership and the three self-rated health distal outcomes, the BCH [46] approach was used, which assesses mean differences across latent profiles while accounting for latent profile classification uncertainty. Missing data is handled using maximum likelihood estimation. Very few participants (1 – 3 cases) were missing data on any indicator.

## Results

The wave 10 sample was 44.4% male and 55.6% female. Two-thirds (62.4%) reported living on a reservation/reserve. The mean annual personal income was $19,537. Most participants were either single (40.0%) or currently living with a romantic partner (41.7%). Fewer participants were married (8.2%), in a relationship and not living with a romantic partner (7.2%), or in some other relationship status (2.9%).

### Class membership

Table 1 displays the latent profile fit statistics. The three information criteria values decreased consistently across models. An elbow plot shows that values start to decrease at a slower rate after a two-class model (not shown). The LMR-LRT test drops from significance at a 3-class model while the BLRT test drops from significance at a 4-class model. Both three- and four-class model produced a low integration profile characterized by low values across all indicators; a high integration profile which was characterized by high values across all indicators; and a moderate integration profile in which values across indicators were somewhere between the low and high integration profiles. The four-class model produced a small class (< 3%) characterized by very low values across all indicators. The three-class model had an entropy value of 0.81, and average latent profile probabilities for most likely latent profile membership ranged from 0.91—0.93, both of which indicate high levels of profile separation and classification accuracy. As such, we retain the more parsimonious three-class model. Ad-hoc analyses show no differences in demographic predictors or self-rated health outcomes for the two “low” integration profiles.

**Table 1:** Latent profile analysis fit statistics

	AIC	BIC	SA-BIC	LMR-LRT	BLRT	Entropy
1 class	31,544.28	31,603.61	31,559.17			
2 classes	30,915.93	31,009.17	30,939.34	631.69*	644.35*	0.81
3 classes	30,685.00	30,812.15	30,716.93	242.07	246.92*	0.81
4 classes	30,510.75	30,671.81	30,551.19	186.52*	190.25*	0.85
5 classes	30,438.80	30,633.77	30,487.75	86.22	87.95	0.85

*AIC* Akaike Information Criteria, *BIC* Bayesian Information Criteria, *SA-BIC* Sample Adjusted BIC, *LMR-LRT* Lo-Mendell-Rubin Likelihood Ratio Test, *BLRT* Bootstrapped Likelihood Ratio Test

^*^p < .05

**Fig. 1:**
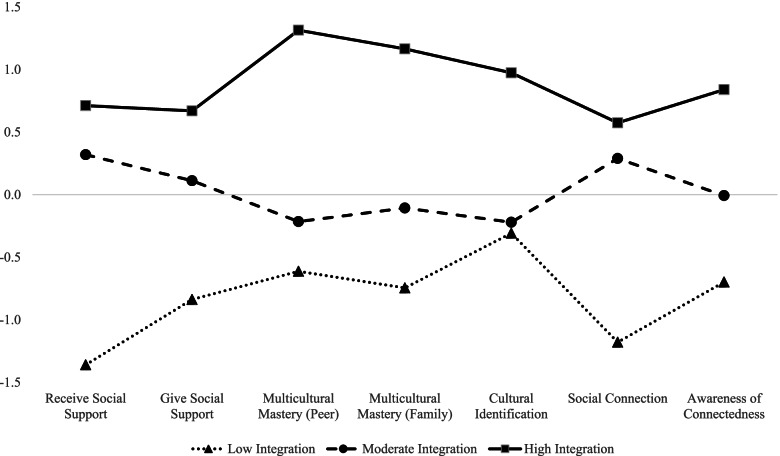
Standardized means (y-axis) for socio-cultural indicators (x-axis). Values above reference line (*M* = 0) indicate values higher than the overall sample mean, and value below the reference line indicate values lower than the overall sample mean

Figure 1 displays the plotted standardized values for each observed indicator within latent profiles. The horizontal reference line represents the overall sample average (Mean = 0) for each indicator. The first latent profile, *low integration* (23.6%), is characterized by values below the overall sample average. The second latent profile, *moderate integration* (56.3%), is characterized by values slightly above or below the sample averages. The third latent profile, *high integration* (20.1%), is characterized by values well above the overall sample average. Ad-hoc ANOVA analyses with Bonferroni multiple comparison tests indicate that, within each indicator, values are significantly different across the three latent profiles (not shown). The only exception to this pattern is for cultural identification, for which means are not significantly different between the low and moderate integration profiles, but both are significantly lower than the high profile.

### Demographic predictors of class membership

Table 2 presents multinomial logistic regression results predicting latent profile membership. Three of the six demographic predictors are significantly associated with latent profile membership. In both the unadjusted and adjusted models, personal income increases the odds of moderate and high integration profile membership, relative to the low integration profile. Personal income does not, however, predict moderate profile membership relative to high integration profile membership. Compared to respondents with less than a high school education, those with a high school diploma/GED, some college, or college degree have higher odds of moderate integration profile membership relative to low integration membership. In adjusted models, however, only, high school diploma/GED remained significant. Compared to respondents with less than a high school education, those whose highest level of education was some college or college graduate have increased odds of high integration membership relative to low integration membership. Compared to respondents with less than a high school education, those whose highest level of education is a college degree have higher odds of high integration profile membership relative to moderate integration membership. Compared to single participants, married participants have higher odds of moderate and high integration profile membership, relative to low integration membership. This relationship, however, drops from significance once all covariates demographics are included. Compared to single participants, those living with a partner have higher odds of moderate and high integration profile membership relative to low integration membership. Being in a non-cohabiting relationship and other relationship status does not predict profile membership. Age, gender, and residing on vs. off reservation/reserve land are not statistically significant predictors of latent profile membership.

**Table 2:** Multinomial logistic regression models predicting latent class membership (*N* = 512)

	Low Integration vs. Moderate Integration		Low Integration vs. High Integration		Moderate Integration vs. High Integration	
	Unadjusted RRR [95% CI]	Adjusted RRR [95% CI]	Unadjusted RRR [95% CI]	Adjusted RRR [95% CI]	Unadjusted RRR [95% CI]	Adjusted RRR [95% CI]
Age	1.02 [0.89, 1.16]	1.02 [0.89, 1.16]	1.07 [0.91, 1.24]	1.05 [0.88, 1.24]	1.05 [0.89, 1.24]	1.03 [0.86, 1.23]
Income	1.05** [1.02, 1.08]	1.03^*^ [1.00, 1.06]	1.06*** [1.03, 1.09]	1.03* [1.00, 1.06]	1.01 [1.00, 1.02]	1.00 [0.98, 1.01]
Female	1.10 [0.66, 1.81]	0.98 [0.56, 1.72]	1.05 [0.59, 1.87]	0.88 [0.45, 1.73]	0.96 [0.57, 1.62]	0.90 [0.51, 1.61]
Resides on Reservation	1.19 [0.71, 2.01]	1.52 [0.83, 2.76]	0.87 [0.49, 1.56]	1.53 [0.78, 3.03]	0.73 [0.43, 1.24]	1.01 [0.56, 1.83]
Education:						
*Less than High School (ref)*						
*High School Diploma or GED*	2.70** [1.45, 5.02]	2.06* [1.04, 4.08]	2.22 [0.94, 5.25]	1.80 [0.74, 4.37]	0.82 [0.43, 1.24]	0.87 [0.37, 2.03]
*Some college*	2.18* [1.08, 4.40]	1.47 [0.63, 3.42]	3.82** [1.60, 9.14]	2.73* [1.05, 7.08]	1.75 [0.75, 4.08]	1.86 [0.79, 4.39]
*College Degree*	4.32** [1.49, 12.56]	2.72 [0.73, 10.06]	17.06*** [5.61, 51.95]	11.72*** [3.15, 43.65]	3.95** [1.67, 9.35]	4.32** [1.69, 11.02]
Marital Status:						
*Single (ref)*						
*Married*	6.21* [1.40, 27.48]	3.93 [0.83, 18.53]	6.13* [1.35, 27.97]	4.11 [0.81, 20.86]	0.99 [0.40, 2.46]	1.04 [0.39, 2.80]
*Living with Partner*	2.78*** [1.56, 4.93]	2.10* [1.15, 3.81]	2.36** [1.24, 4.50]	2.00 [0.99, 4.05]	0.85 [0.47, 1.53]	0.95 [0.50, 1.80]
*In Relationship*	1.82 [0.68, 4.86]	1.29 [0.42, 3.96]	1.18 [0.35, 4.00]	1.09 [0.31, 3.79]	0.65 [0.21, 2.04]	0.84 [0.26, 2.78]
*Other*	1.67 [0.40, 6.94]	1.26 [0.34, 4.65]	0.86 0.12, 6.04]	0.49 [0.06, 4.07]	0.51 [0.08, 3.34]	0.39 [0.05, 3.14]

*RRR* Relative risk ratio, *CI* Confidence interval

^*^p < .05; ^**^p < .01; ^***^p < .001

### Health outcomes across class membership profiles

Table 3 displays the distal self-rated health outcomes across latent profile membership. Latent profiles are significantly associated with self-rated physical, mental, and spiritual health. For each outcome, means are lowest in the low integration profile and highest in the high integration profile. Means in the moderate integration are in between the low and high integration means. Within each of these distal outcomes, all means are statistically significantly different from one another.

**Table 3:** Self-rated health means by socio-cultural integration latent profile membership

	Low Integration	Moderate Integration	High Integration	χ^2^ Test
Physical Health	1.74_a_	2.22_b_	2.60_c_	37.61^***^
Mental Health	1.75_a_	2.27_b_	2.71_c_	51.52^***^
Spiritual Health	1.79_a_	2.15_b_	2.69_c_	40.27^***^

For each outcome, means that do not share a subscript are significantly different (*p* < .05)

^*^p < .05; ^**^p < .01; ^***^p < .001

## Discussion

One goal of this study was to address prior limitations in the assessment of broad indicators of socio-cultural integration. We examined multi-dimensional indicators of socio-cultural integration, identified patterns of responses to these indicators using LPA, and compared the relationships of these patterns to wellbeing among a sample of Indigenous young adults. While several of the individual constructs indicative of socio-cultural integration used in this study have been linked to better health in prior research [2, 29, 38, 47], the current LPA results suggest that the totality of socio-cultural integration variables may coalesce into something greater than the sum of its parts. This is further exemplified in that: a) those in the high integration class (high in each indicator examined) had the best self-rated health outcomes compared to the other two groups while those low across each indicator had the worst outcomes; and b) each of the 7 socio-cultural integration measures were significantly different from one another across the 3 latent profiles with one exception: reports of cultural identification were similar for those in the low and moderate integration groups. The fact that socio-cultural integration was positively associated with holistic indicators of health (physical, mental, spiritual) is notable in light of Indigenous conceptions of health that are inclusive of each of these domains, an orientation increasingly supported by empirical evidence as critical to fully understanding and promoting wellbeing [48].

Our results support calls for expanded operationalization of socio-cultural integration factors, a goal that aligns with Indigenous worldviews emphasizing inter-relationality of these concepts [13, 49, 50]. Broadened assessment of social connections to include emphasis on *culture* is particularly important given Tribal priorities to preserve and revitalize cultural values in the face of widening health inequities and in attempt to redress the ongoing impact of historical trauma and colonization [25]. Encouraging wellness via community and cultural connections are acts of healthy Tribal Nation building, self-determination, and sovereignty [50]. Amidst these endeavors is growing attention to Indigenization, or the inclusion and prioritization of Indigenous perspectives and worldviews in research, policy, and practice [51]. Our findings suggest that an Indigenized approach to conceptualizing and assessing socio-cultural integration may well advance understanding of the protective influence of this construct. Relatedly, decolonization processes involve deconstructing privileged, oppressive, often Euro-centric worldviews that perpetuate values like individualism, domination, and capitalism while valuing, reclaiming, and lifting up Indigenous ways of knowing and being [52, 53]. Decolonizing approaches compel collective involvement, emphasize relationships, and require critical self-examination [52]. In direct relation to our findings, decolonization addresses disconnection: “instead of divide, control, exploit, we embrace a new paradigm of connect, relate, belong” ([54]; p 34).

Our analyses also examined demographic predictors of socio-cultural integration. Prior research suggests differences in integration across demographic factors, but these processes are not completely clear. For instance, a meta-analysis demonstrated higher social integration among college students from middle (versus working class) backgrounds; however, the type of integration factors measured moderated this relationship. More specifically, effects of social class on social integration where larger when assessing college-specific interpersonal/societal contexts compared to general social activities (whether formal and informal) [7]. Relatedly, a sizeable body of research has indicated that married individuals report better health than their unmarried counterparts, in part due to social support, but this relationship varies substantially across contexts, [55] and there is conflicting evidence of heightened social ties among single individuals compared to married or previously married persons [56]. In the current study, we found no differences in socio-cultural integration profile membership by gender, age, or residing on/off reservation/reserve status. Differences were observed across profiles by income, education, and relationship status; however, consistent with prior research, these distinctions were not always robust, particularly after adjusting for other demographic variables. The one exception to this result was found among college graduates, who were significantly more likely to belong to higher socio-cultural integration groups. This suggests that education promotes, rather than discourages, socio-cultural integration for the Indigenous young adults in our sample. Notably, exploring the *type* and *form* of education experienced by participants would be a worthy future endeavor. For instance, there are likely differences for students attending schools that celebrate Native identity (e.g., Tribal Colleges and schools) compared to other predominantly White institutions in which Native contributions may be diminished, ignored, or even punished [57, 58].

This study leaves room for other areas of inquiry as well. Indigenous wellbeing models include factors beyond those we were able to measure, such as intergenerational connections and ancestral kinship. For instance, one community-based author for this manuscript, S.W., notes the profound influence of recognizing the sacrifices and contributions of our Ancestors. This awareness of connection is thus a driver of positive choices for one’s self and in consideration of future generations: being a part of this perpetual cycle gives purpose for living. In addition, we explored direct associations between socio-cultural integration and health; however, stress-buffering or mediating processes are also plausible [2, 59] and important for future investigation. Our quantitative models do not permit exploration of the lived experiences leading to high or low ratings of social and cultural connection; such qualitative perspectives could yield critical insights on how to promote these protective factors with communities.

## Conclusions

Overall, the results of this study support the notion that community, culture, and a sense of connection are significant correlates of wellness for this sample of Indigenous young adults. Such findings underscore the importance of decolonizing approaches to address social isolation and promote health integration. From a research and public health programming standpoint, the very process of decolonizing becomes an act of inclusion, community connection, and cultural reclamation [60]. Authentic partnership models, including CBPR, can serve as catalysts or supports for community action to engage programs and activities that promote positive cultural identity, social connectivity, and emphasize our interconnections with others and the environment around us [13, 61]. In short, the indicators of socio-cultural integration measured in the current study represent malleable health promotion factors best encouraged through decolonization. To that end, promoting these factors is one step forward in alliance with Indigenous ontologies founded upon recognition of *all relations.*

## Data Availability

The datasets generated and/or analyzed during the current study are not publicly available due to data ownership agreements with participating Tribes. Each individual Tribal Government owns the data corresponding to participants from within its jurisdiction, and each Tribal Government withholds to right to respond to requests for access to data, which can be directed to the PI for the project, Dr. Melissa Walls (mwalls3@jhu.edu).

## Abbreviations

LPA: Latent profile analysis
CBPR: Community-based participatory research
AIC: Akaike information criteria
BIC: Bayesian information criteria
SA-BIC: Sample adjusted BIC
LMR-LRT: Lo-Mendell-Rubin likelihood ratio test
